# Synthesis of Multi-Functional Nano-Vectors for Target-Specific Drug Delivery

**DOI:** 10.3390/polym13030451

**Published:** 2021-01-30

**Authors:** Tzu-Chien Wu, Pei-Yuan Lee, Chiao-Ling Lai, Chian-Hui Lai

**Affiliations:** 1 Graduate Institute of Biomedical Engineering, National Chung Hsing University, Taichung 402, Taiwan; s106035023@mail.nchu.edu.tw (T.-C.W.); g107068001@mail.nchu.edu.tw (C.-L.L.); 2 Orthopedic Department, Show Chwan Memorial Hospital, Changhua 500, Taiwan; ADS275@show.org.tw

**Keywords:** magnetic nanoparticle (MNP), drug delivery, dual-responsive nano-carrier, tumor microenvironment (TME), enhanced permeability and retention effect (EPR effect)

## Abstract

Magnetic nanoparticles have gained attention in cancer therapy due to their non-toxic properties and high bio-compatibility. In this report, we synthesize a dual-responsive magnetic nanoparticle (MNP) that is sensitive to subtle pH and temperature change as in the tumor microenvironment. Thus, the functional doxorubicin (DOX)-loaded MNP (DOX-PNIPAM-PMAA@Fe_3_O_4_) can perform specific DOX releases in the cancer cell. The particle was characterized by scanning electron microscopy (SEM), dynamic light scattering (DLS), zeta-potential, Fourier-transform infrared spectroscopy (FTIR), and thermogravimetric analysis (TGA). The microscopy data revealed the particle as having a spherical shape. The zeta-potential and size distribution analysis data demonstrated the difference for the stepwise modified MNPs. The FTIR spectrum showed characteristic absorption bands of NH_2_-SiO_2_@Fe_3_O_4_, CPDB@Fe_3_O_4_, PMAA@Fe_3_O_4_, and PNIPAM-PMAA@Fe_3_O_4_. Drug-loading capacity and releasing efficiency were evaluated under different conditions. Through an in vitro analysis, we confirmed that PNIPAM-PMAA@Fe_3_O_4_ has enhanced drug releasing efficiency under acidic and warmer conditions. Finally, cellular uptake and cell viability were estimated via different treatments in an MDA-MB-231 cell line. Through the above analysis, we concluded that the DOX-loaded particles can be internalized by cancer cells, and such a result is positive and prospective.

## 1. Introduction

To attain specific drug delivery while maintaining drug stability has been a significant issue in the research field of tumor therapy [1,2]. Modified magnetic nanoparticles (MNPs) have been reported to act as a platform for drug delivery that could overcome drug degradation and cytotoxicity problems [3,4,5]. Tumor cells have greater glycolytic activity, and the corresponding metabolites lower the pH value of tumor sites; on the other hand, the replication and proliferation processes may produce heat and increase the temperature in the tissue [6,7]. Based on the above-mentioned aspects, recent research has pointed out several innovations to utilize these abnormal features of the tumor microenvironment (TME) [8,9,10,11,12]. For instance, modified MNPs have been used as a vector for drug delivery due to their low cytotoxicity and high bio-compatibility [5]. Through modification of the particle surface, particles can respond to subtle environment changes and, thus, could be able to perform target-specific drug delivery [13,14].

Superparamagnetism is a unique characteristic of magnetic nano-materials. Owing to this feature, magnetic nanoparticles are an easy-to-operate material while being highly bio-compatible, highly stable, and less cytotoxic. Therefore, there is no doubt that the material can be applied in the biomedical field, such as for MRI image improvement, tumor magneto-therapy, and bio-separation [15,16,17]. As for the synthesis method, in addition to cobalt and nickel, most were formed with Fe_3_O_4_ iron-cored via co-precipitation. Co-precipitation is a synthesis method that features great yield and gentle reaction condition [18].

In order to achieve specific targeting to TME, we designed a nanovector for target-specific drug delivery. TMEs in blood vessels, immune cells, fibroblasts, various signaling molecules, and extracellular matrix are different from healthy tissue. Tumor cells obtain large amounts of oxygen and nutrition for rapid growth and cells may secrete vascular endothelial growth factor (VEGF) and facilitate angiogenesis [19,20]. Tumor cells have complicated interactions and signaling transduction to enhance carcinoma metastasis, growth, and inflammation [6,21]. Tumor-associated macrophages (TAMs) are immune cells in the TME and can be divided into M1 and M2 types. M1-type TAMs can express and produce pro-inflammatory cytokines to induce anti-tumor responses. M2-type TAMs, on the contrary, secrete anti-inflammatory agents such as transforming growth factor-β (TGF-β), interleukin-10 (IL-10), and epidermal growth factor, which inhibit immune responses and induce angiogenesis and carcinoma metastasis [22]. Nanoparticles and macromolecular drugs may remain in the TME through an enhanced permeability and retention effect (EPR effect). Cancer tissues usually consist of abnormal blood vessels and poorly aligned defective endothelial cells, where inter-endothelial junctions of tumors lie in the range of 40–80 nm while the inter-endothelial junction of normal endothelial cells is 8 nm [23]. In addition, cancer cells lack effective lymphatic drainage. Therefore, nanoparticles and macromolecular drugs tend to accumulate at tumor tissues rather than normal tissues due to the EPR effect. When tumor size reaches 200 μm, the TME forms and induces the EPR effect [19,20]. We have used this feature and developed nanovectors to target cancer cells [23,24].

In this study, the reverse addition-fragmentation chain transfer (RAFT) polymerization method is used for MNP modifications. RAFT is a common radical polymerization mediated by a RAFT agent to achieve radical transfer and chain elongation. In this kind of polymerization, certain dithioester derivatives serve as the RAFT agent, and azobisisobutyronitrile (AIBN) acts as a radical initiator to activate radical formation, propagation, RAFT pre-equilibrium, re-initiation, main RAFT equilibrium, and termination [25]. RAFT polymerization features a broad application of monomers, gentle condition, and narrow polydispersity index (PDI), traits that are key to modifying functionalized nanoparticles [12,26].

To date, several cancer-specific responsive nanoparticles have been developed [27]. For example, a silica nanoparticle was designed to be both pH- and thermo-responsive via RAFT polymerization [27]. When the nanoplatform was subjected to low pH values and high temperature, carboxylate groups on the surface protonated to form the COOH group. In addition, when *N*-isopropylacrylamide (NIPAM) aggregates above the lower critical solution temperature (LCST), it results in a synergistic effect of doxorubicin (DOX) releasing. The formation of the COOH group would weaken the electrostatic attraction between drugs and particle surface. Furthermore, when the reaction temperature is higher than the LCST of NIPAM, NIPAM on the particle surface would be insoluble, resulting in aggregation of MNPs that spread DOX. This finding definitely improved the low effect on drug releasing of temperature that was reported in the previous literature.

**Scheme 1:**
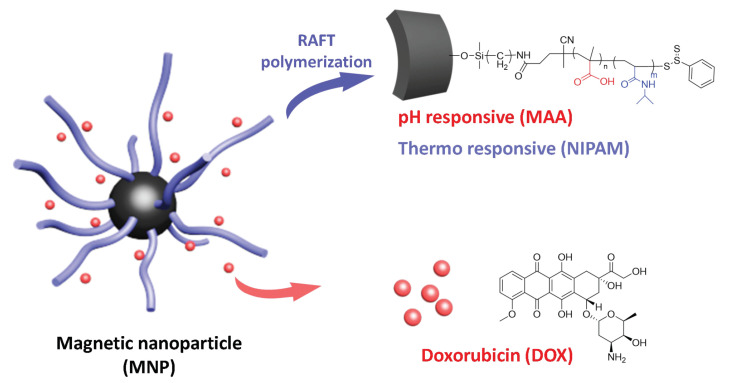
Overall structure of the nanoparticle design.

This series of experiments was designed based on the above-mentioned literature review. We aimed to synthesize Fe_3_O_4_-cored nanoparticles via co-precipitation and utilize the sol-gel method to complete amine functionalization. RAFT polymerization is taken to modify the particle surface with responsive moiety. Doxorubicin, an anti-tumor drug, is then loaded onto the particle and subjected to in vitro drug release (Scheme 1).

## 2. Materials and Methods

### 2.1. Material

FeCl_3_ (Alfar Aesar), FeCl_2_·4H_2_O (Alfar Aesar), tetraethyl orthosilicate (TEOS, Sigma-Aldrich, St. Louis, MO, USA), 3-aminopropyltrimethoxysilane (APS, Alfar Aesar, Haverhill, MA, USA), 2-propanol (MACRON, Radnor, PA, USA), ammonium hydroxide solution (25%, MACRON), 4-nitrobenzaldehyde (NPC, Alfar Aesar), triethyl amine (TEA, Alfar Aesar), ethanolamine (Sigma-Aldrich), 4-Cyano-4-(phenylcarbonothioylthio)pentanoic acid (CPDB, STREM, Newburyport, MA, USA), 1-Ethyl-3-(3-dimethylaminopropyl)carbodiimide (EDC, Sigma-Aldrich), *N*-hydroxysuccinimide (NHS, TCI, Tokyo, Japan), methacrylic acid (MAA, ACROS), azobisisobutyronitrile (AIBN, UniRegion Bio-Tech), ethanolamine (Sigma-Aldrich), *N,N*-Diisopropylethylamine (DIPEA, TCI), Dimethyl formaldehyde (DMF, DUKSAN), tetrahydrofuran (THF, DUKSAN, Gyeonggi-do, Korea), dimethyl sulfoxide (DMSO, Fisher), and 3-(4,5-dimethylthiazol-2-yl)-2,5-diphenyltetrazolium bromide (MTT, Sigma) were used as received. Analytical thin-layer chromatography (TLC) was performed using pre-coated plates (Silica Gel 60 F_254_, Merck, Kenilworth, NJ, USA).

A UV–Vis spectrum reader (BioTek, SYNERGY Mx, Winooski, VT, USA), a fluorescence spectrometer (Leica DMI4000 B, Wetzlar, Germany), dynamic light scattering (DLS) (HORIBA, SZ-100, Kyoto, Japan), FTIR (Perkin Elmer Intruments, Spectrum One, Waltham, MA, USA), TGA/DSC (Mettler-Toledo, 2-HT, Columbus, OH, USA), a centrifuge (HERMLE, Gosheim, Germany), and a fluorescence microscope (HORIBA, Fluorolog, Kyoto, Japan) were used for analytical purposes.

### 2.2. Preparation of HO@Fe_3_O_4_

The particle HO@Fe_3_O_4_ was synthesized by co-precipitation of FeCl_3_ and FeCl_2_·4H_2_O under 1.5 N NaOH_(aq)_. FeCl_3_ (2.0 g, 12.3 mmol) and FeCl_2_·4H_2_O (5.2 g, 26.2 mmol) were mixed in 25 mL of 12.1 N HCl. The mixture was then added dropwise into 250 mL of 1.5 N NaOH. The black precipitate was collected after 4000 rpm centrifugation for 15 min. Then, 500 mL of 0.01 N HCl solution was added into the collected residues under vigorous vortex. HO@Fe_3_O_4_ was then obtained and washed with deionized water.

### 2.3. Preparation of NH_2_-SiO_2_@Fe_3_O_4_

An amine moiety was attached to HO@Fe_3_O_4_ by a sol-gel method that used tetraethyl orthosilicate (TEOS) and 3-aminopropyltrimethoxysilane (APS). HO@Fe_3_O_4_ (500 mg) and TEOS (0.42 mL) were mixed in 11 mL of 2-propanol. Ammonium hydroxide (1.58 mL) was then added. The flask was placed in an oil bath at 55 °C for 2 h. Then, 0.42 mL of APS was added into the above mixture and reacted for 15 h. The final product was washed twice by 2-propanol and deionized water consecutively and then dried under vacuum.

### 2.4. Quantification of Amine Functional Group

NH_2_-SiO_2_@Fe_3_O_4_ (5.0 mg) was suspended in 1.0 mL of MeOH and then subjected to sonication for 30 min. The particles were washed using THF and DCM three times each. Next, the particles were treated with 10 mg of 4-nitrobenzaldehyde (NPC) and 10 μL trimethylamine (TEA) for 1 h. NPC@Fe_3_O_4_ was then obtained and washed thrice using DCM. MeOH (1 mL), 10 μL ethanolamine, and 10 μL TEA were added into the flask and left for 30 min. A 4-nitrobenzaldehyde solution was obtained via hydrolysis followed by ethanolamine and was subsequently estimated by comparison of the calibration curve. The results showed that the amine concentration attained was around 120 nmol per milligram of particle.

### 2.5. Attachment of RAFT Agent to Particle Surface

NH_2_-SiO_2_@Fe_3_O_4_ (5 mg) was dissolved in DMF. EDC (11.50 mg, 0.06 mmol), NHS (6.91 mg, 0.06 mmol), DIPEA (3.2 μL, 0.06 mmol), and CPDB (16.76 mg, 0.06 mmol) were added to the above solution and placed at room temperature for 16 h. The product CPDB@Fe_3_O_4_ was then obtained after washing twice with DMF and dried under vacuum.

### 2.6. Synthesis of PMAA@Fe_3_O_4_

CPDB@Fe_3_O_4_ (10.4 mg, 1.25 × 10^−3^ mmol) and methacrylic acid (MAA, 275 mg, 3.2 mmol) were dissolved in 1 mL THF in a 10-mL flask. AIBN (32.5 μg, 198 μmol) was then added. The mixture was then degassed by the typical three freeze–pump–thaw cycle. After being backfilled with argon, the flask was placed in an oil bath at 60 °C for 3 h. The RAFT polymerization was quenched by 1 mL ether in an ice bath.

### 2.7. Synthesis of PNIPAM-PMAA@Fe_3_O_4_

PMAA @Fe_3_O_4_ (23.4 mg) and *N*-Isopropylacrylamide (NIPAM, 158.4 mg, 1.4 mmol) were dissolved in 1 mL DMF. AIBN (23 μg, 140 μmol) was then added. The mixture was then degassed by the typical three freeze–pump–thaw cycle. After being backfilled with argon, the flask was placed in an oil bath at 80 °C overnight. The RAFT polymerization was quenched by 1 mL ether in an ice bath. The PNIPAM-PMAA@ Fe_3_O_4_ was dried under vacuum for the next experiment.

### 2.8. Preparation of DOX-PNIPAM-PMAA@Fe_3_O_4_

PNIPAM-PMAA@Fe_3_O_4_ (1.5 mg) and DOX (500 μg) were mixed in 1 mL PBS, pH 8.5. The mixture was placed at room temperature overnight. After ultracentrifugation at 10,000 rpm for 30 min, the supernatant was collected and subjected to UV–Vis analysis. The mass of DOX attached to magnetic nanoparticles was estimated by comparing with the standard curve.

### 2.9. UV-Vis Absorption Measurement for Drug Loading

Doxorubicin has a significant peak at the 480 nm endpoint. Therefore, the supernatant of the DOX-PNIPAM-PMAA@Fe_3_O_4_ sample was collected to estimate the drug loading content (DL contents, %) and drug encapsulation efficiency (drug EE, %).

### 2.10. DLS and Zeta-Potential Measurement

Samples of HO@Fe_3_O_4_ (1 mg), NH_2_-SiO_2_@Fe_3_O_4_ (1 mg), CPDB@Fe_3_O_4_ (1 mg), PMAA@Fe_3_O_4_ (1 mg), and PNIPAM-PMAA@Fe_3_O_4_ (1 mg) were collected and dried under high vacuum. Particles of HO@Fe_3_O_4_, NH_2_-SiO_2_@Fe_3_O_4_, and PNIPAM-PMAA@Fe_3_O_4_ were dispersed in 2 mL dd-H_2_O and sonicated for 30 min. Then, the solution was subjected to DLS analysis (HORIBA, SZ-100). The same method was employed for CPDB@Fe_3_O_4_ and PMAA@Fe_3_O_4_ but the particles were dispersed in DMF for better polydiseperse Index (PDI). For zeta-potential analysis, each sample (1 mg) was collected and dispersed in dd-H_2_O. After sonication for 30 min, the samples were subjected to zeta-potential analysis.

### 2.11. Drug Loading Analysis Through Fluorescence Spectrometer

According to the literature, doxorubicin (DOX) has a significant fluorescence emission signal at 560 and 590 nm under 480-nm excitation. As a result, DOX-PNIPAM-PMAA@Fe_3_O_4_ was dissolved in H_2_O and an analysis was conducted using a fluorescence spectrometer to further ensure whether doxorubicin was loaded onto the particle surface.

### 2.12. In Vitro DOX Release from Magnetic Nanoparticles

DOX-PNIPAM-PMAA@Fe_3_O_4_ (1.5 mg) was washed twice with H_2_O. DOX-PNIPAM-PMAA@Fe_3_O_4_ was well dispersed in different acidic conditions in phosphate-buffered saline (PBS) buffer (pH 7.0, 5.0, or 2.5) and placed at 37 °C for a period of time. Each sample was collected at certain time points and subjected to UV–Vis analysis to estimate the mass of total DOX released from the particles. In the same method, DOX-PNIPAM-PMAA@Fe_3_O_4_ (1.5 mg) was well dispersed in phosphate-buffered saline (PBS) buffer (pH 2.5) and placed at room temperature and 40 °C. Each sample was collected and subjected to UV–Vis analysis to estimate DOX release efficiency.

### 2.13. Cell Viability Analysis via MTT Assay

The MDA-MB-231 cell line was used as the cell material for the cell viability analysis. Cells were incubated in a 96-well microplate overnight (10^4^ cells/well). PNIPAM-PMAA@Fe_3_O samples were acquired through sequencing dilution (0.1 and 0.02 mg/mL for particle concentration). DOX-PNIPAM-PMAA@Fe_3_O_4_ (0.1 and 0.02 mg/mL for particle concentration) samples were obtained through the same method (DOX concentration was 5.4 and 1.1 μg/mL for each, respectively). MDA-MB-231 cells were treated with the above-mentioned samples and PBS (control) for three replications. After 24 h, particles were washed away twice with PBS and further placed at 37 °C to incubate for 48 and 72 h. After MTT addition for 4 h, purple crystallization was dissolved with DMSO and subjected to UV-Vis absorption analysis under 570-nm excitation.

### 2.14. Cellular Uptake Analysis via Microscopy

MBA-MB-231 cells were plated previously in a 12-well plate (2 × 106 cell per well). Cells were treated with free DOX (5 and 1 μg/mL), DOX-PNIPAM-PMAA@Fe_3_O_4_ (5 and 1 μg/mL for DOX-loaded concentration; 0.1 and 0.02 mg/mL for particle concentration), and PBS as control. All the treatments lasted for 4 h. After 4 h of incubation, particles were washed away with PBS. Then, each well was observed under a microscope.

## 3. Results

### 3.1. Synthesis of DOX-PNIPAM-PMAA@Fe_3_O_4_

**Scheme 2:**
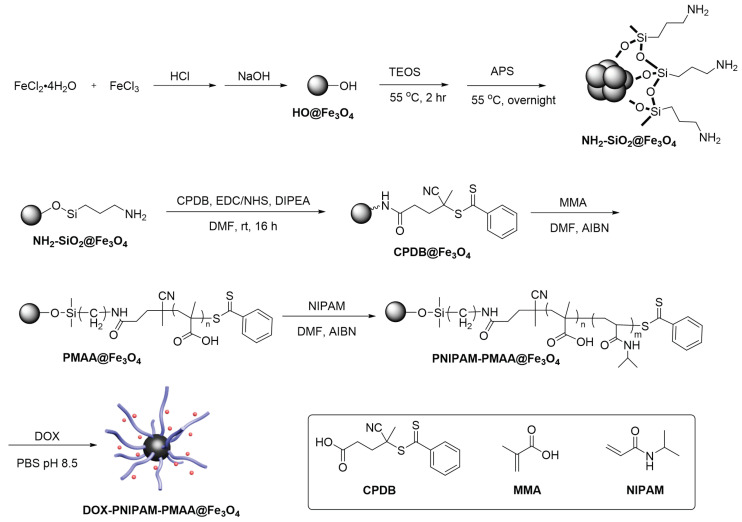
Synthesis scheme of doxorubicin (DOX)-loaded dual-responsive nanoparticles (DOX-PNIPAM-PMAA@Fe_3_O_4_).

The iron-cored particle (HO@Fe_3_O_4_) was prepared by the co-precipitation method and then followed by the two-step sol-gel method to yield NH_2_-SiO_2_@Fe_3_O_4_ (Scheme 2). In this series of experiments, 4-Cyano-4-(phenylcarbonothioylthio)pentanoic acid (CPDB) was utilized as a RAFT agent. The attachment reaction of CPDB to NH_2_-SiO_2_@Fe_3_O_4_ was processed by EDC/NHS activation to yield CPDB@Fe_3_O_4_. The CPDB@Fe_3_O_4_ was subsequntly followed by RAFT polymerization to polymerize the MAA and NIPAM monomers onto the particle surface. Finally, the PNIPAM-PMAA@Fe_3_O_4_ was incubated with doxorubicin under basic conditions for 24 h to yield the final product, DOX- PNIPAM-PMAA@Fe_3_O_4._

### 3.2. SEM and DLS Analysis

**Figure 1:**
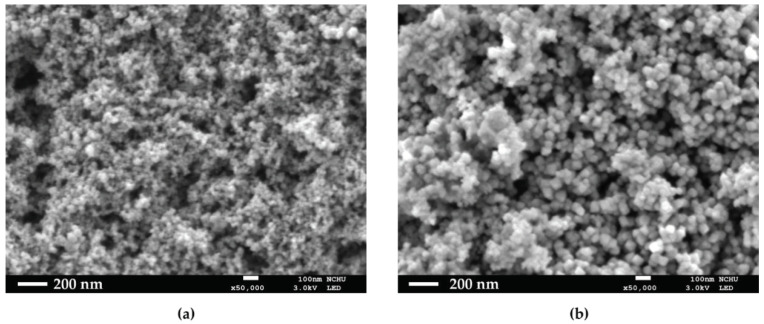
SEM images of (**a**) HO@Fe_3_O_4_ and (**b**) NH_2_-SiO_2_@Fe_3_O_4_.

**Figure 2:**
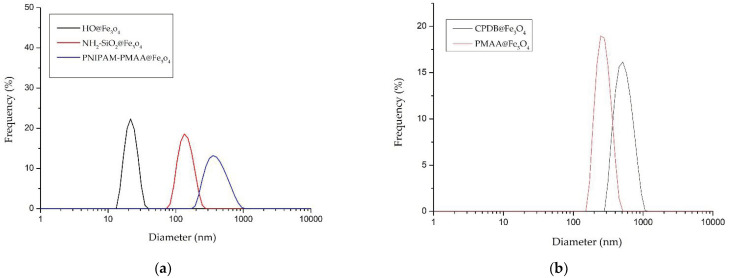
Distribution pattern of the hydrodynamic diameters of (**a**) HO@Fe_3_O_4,_ NH_2_-SiO_2_@Fe_3_O_4_ and PNIPAM-PMAA @Fe_3_O_4_ in dd-H_2_O and (**b**) CPDB@Fe_3_O_4,_ and PMAA@Fe_3_O_4_ in dimethyl formaldehyde (DMF).

HO@Fe_3_O_4_ and NH_2_-SiO_2_@Fe_3_O_4_ were observed under SEM to determine their structures. As shown in Figure 1a,b, both HO@Fe_3_O_4_ and NH_2_-SiO_2_@Fe_3_O_4_ are spherical-shaped, and amine-functionalized particles have an evidently larger size compared with HO@Fe_3_O_4_. As shown in the DLS pattern (Figure 2, Table 1), the hydrodynamic diameter of HO@Fe_3_O_4_ is around 20 nm. After amine functionalization, the size of particles increased to around 300 nm. Particle sizes modified with CPDB and MAA were analyzed in DMF since they were not well dispersed in water. Compared to the above-mentioned particles, particles modified with CPDB, MAA, or NIPAM did not seem to increase in size as their hydrodynamic diameters roughly ranged from 200 to 500 nm.

### 3.3. Zeta Potential Analysis

**Figure 3:**
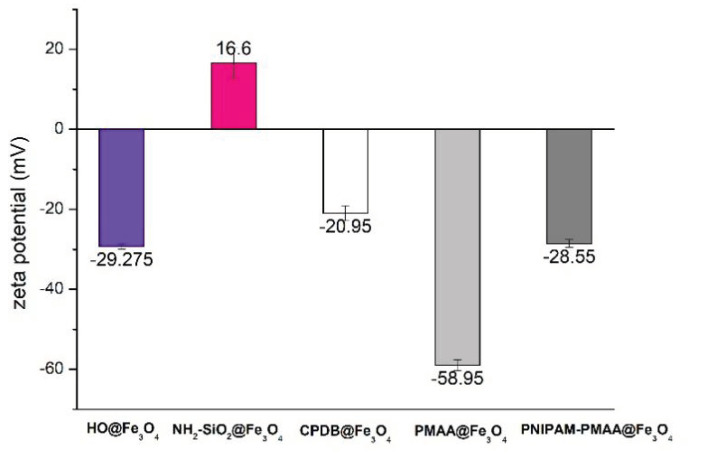
Zeta-potential analysis of HO@Fe_3_O_4_, NH_2_-SiO_2_@Fe_3_O_4_, CPDB@Fe_3_O_4_, PMAA@Fe_3_O_4_, and PNIPAM-PMAA@Fe_3_O_4_ in dd-H_2_O.

As shown in Figure 3, Fe_3_O_4_-cored magnetic nanoparticles are negatively charged on the surface owing to hydroxyl groups, while amine-functionalized particles are positively charged on the surface because of the amine groups. After modification of MAA, the carboxyl group on the surface would partly deprotonate to form COO^−^, which causes the charge on the particle surface to be more negative. Afterward, the modification of NIPAM causes the charge distribution on the surface to be less negative due to polymerization of more positively charged monomers. Based on the aforementioned aspects, it can be inferred that MAA and NIPAM were successfully polymerized onto the particles.

### 3.4. Fourier-Transform Infrared Spectroscopy (FTIR) and Thermogravimetric Analysis (TGA)

**Figure 4:**
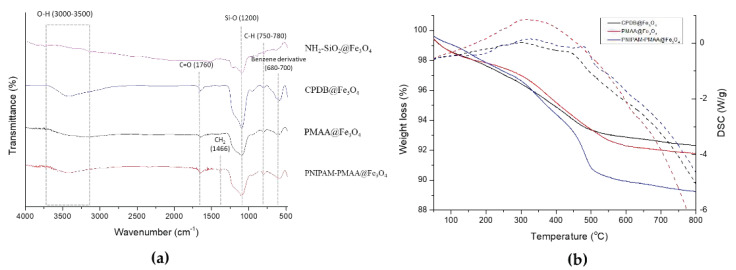
(**a**) FTIR spectra of HO@Fe_3_O_4_, NH_2_-SiO_2_@Fe_3_O_4_, CPDB@Fe_3_O_4,_ PMAA@ Fe_3_O_4_, and PNIPAM-PMMA@Fe_3_O_4_ and (**b**) thermogravimetric analysis (TGA) of CPDB@Fe_3_O_4_, PMAA@ Fe_3_O_4_ and PNIPAM-PMMA@Fe_3_O_4._ The dashed lines indicate the heat flow variation of CPDB@Fe_3_O_4_, PMAA@ Fe_3_O_4_, and PNIPAM-PMMA @Fe_3_O_4_, respectively.

NH_2_-SiO_2_@Fe_3_O_4_, CPDB@Fe_3_O_4_, PMAA@Fe_3_O_4_, and PNIPAM-PMAA@Fe_3_O_4_ were analyzed via FTIR. As shown in Figure 4, NH_2_-SiO_2_@Fe_3_O_4_ can be identified through the signal presence of the Si-O bond at 1200 nm. CPDB@Fe_3_O_4_, PMAA@Fe_3_O_4_, and PNIPAM-PMAA@Fe_3_O_4_ can be identified through the presence of the carbonyl group (C=O) at 1760 nm (stretching) and the benzene derivative signal at around 680–700 nm, inferring the attachment of the RAFT agent. Besides, the pattern of PNIPAM-PMAA@Fe_3_O_4_ also contains a sharp signal at 1466 nm (bending), indicating the presence of CH_2_. Particles modified with monomers were characterized through thermogravimetric analysis (TGA). CPDB@Fe_3_O_4_ and PMAA@Fe_3_O_4_ had subtle significant differences in the TGA graphs. The weight loss of CPDB@Fe_3_O_4_ and PMAA@Fe_3_O_4_ was 92% and 91%, respectively. The 1% loss indicates the attachment of the MAA. The pattern of PNIPAM-PMAA@Fe_3_O_4_ was similar to the above-mentioned polymers, but an obvious steep reduction could be seen when heated up to 400–500 ^°^C and a further loss to the mass to 89%. Through the slight variations, we could confirm that NIPAM was polymerized onto the particles. In Figure 4b, we also analyze the differential scanning calorimetry (DSC) of PNIPAM-PMAA@Fe_3_O_4_.

### 3.5. Naked-Eye Observation of MNP Physical Properties

The processing and preparation of the above-mentioned analysis were relatively complicated and time-consuming. To quickly ensure whether monomers were polymerized onto the particle surface, we introduced a simple method. Due to different physical properties on particles, we concluded their suspension conditions in different solvents (Table 2). First, CPDB@Fe_3_O_4_ was well suspended in H_2_O, but after a few minutes, precipitation occurred. This phenomenon can be attributed to the benzene functional group on the surface that makes it less hydrophilic. Secondly, PNIPAM-PMAA@Fe_3_O_4_ seemed difficult to separate by the magnetic field and tended to precipitate in DCM. Such phenomena indicated that N-isopropylacrylamide monomers polymerized on the particles, increasing solubility in DCM.

### 3.6. Drug Loading Capacity Analysis

**Figure 5:**
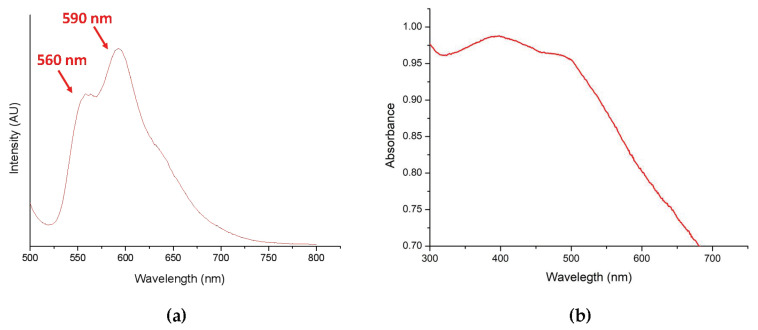
(**a**) Fluorescence emission spectrum of DOX-loaded particle (DOX-PNIPAM-PMAA@Fe_3_O_4_) suspension (excitation 480 nm); (**b**) UV–Vis absorption spectrum of DOX-PNIPAM-PMAA@Fe_3_O_4._

A standard curve was obtained by dissolving DOX in PBS at pH 8.5 and subjected to UV-Vis absorbance analysis (480 nm) after serial dilution. After incubation in PBS, pH 8.5, supernatant was collected to estimated drug loading content (DL content, %) and drug encapsulation efficiency (drug EE, %). Afterwards, DL content and drug EE were evaluated to be 12.1% and 43.6%, respectively. Furthermore, DOX-loaded nanoparticles (DOX-PNIPAM-PMAA@Fe_3_O_4_) were analyzed using a fluorescence spectrometer. We found that under 480-nm excitation, two obvious signals (at 560 and 590 nm) were observed and this was inferred to be the effect from doxorubicin (Figure 5a). We also investigated the UV-Vis absorption of DOX-PNIPAM-PMAA@Fe_3_O_4_ (Figure 5b). Through the graph, two subtle peaks were observed at roughly 400 and 500 nm. Unlike the representative signal of DOX in the UV-Vis absorption spectrum, it was hard to analyze the spectroscopic property of the DOX-loaded particle. We inferred that the phenomenon arose from the interference of the iron oxide core.

### 3.7. In Vitro Drug Releasing Efficacy Analysis

**Figure 6:**
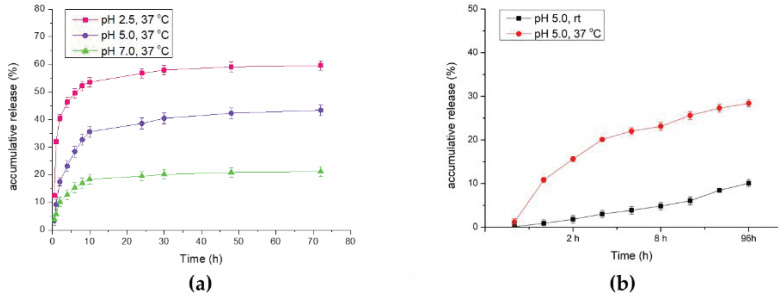
In vitro drug releasing efficacy analysis through UV–Vis analysis. (**a**) Comparison of accumulative release rate under different pH values. (**b**) Comparison of accumulative release rate at different temperatures.

All samples were collected and subjected to UV-Vis absorption analysis. The results are summarized in Figure 6a. As shown in Figure 6, under extreme reaction conditions (pH 2.5), DOX was released swiftly in the first 30 min, and the accumulative release percentage reached nearly 59% after 72 h of treatment. In the neutral reaction condition (pH 7.0), the DOX-releasing efficacy did not seem optimal, which only reached 21% of all the drug loaded. Afterwards, in the modest acidic reaction environment (pH 5.0), DOX releasing had the most optimal results. Under the condition of pH 5.0, the cumulative release percentage (43%) could not compete with the result of pH 2.5, but it had relatively stable and mild releasing rates, which was consistent with our expectation that in the tumor microenvironment (at around pH 5.0–6.5), acidic conditions could promote the carboxyl group on polymer to protonate, which results in effective drug release. In Figure 6b, we investigate the variation at different temperatures. At the identical pH 5.0 condition, we found that higher temperature (37 °C) could successfully enhance the net release of doxorubicin (28%) in comparison with the release rate at room temperature (10%).

### 3.8. Cell Viability Analysis via MTT Assay

**Figure 7:**
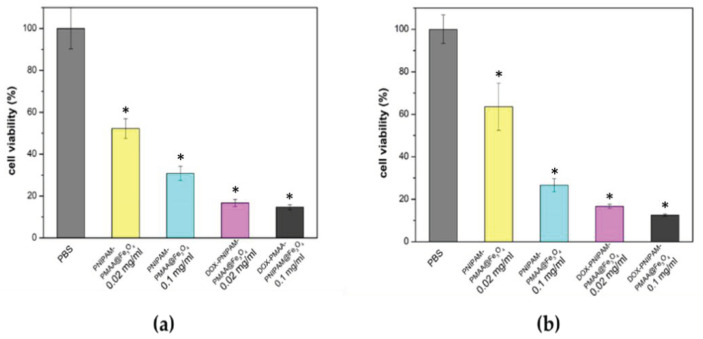
Cell viability analysis. (**a**) Cell viability analysis of the MDA-MB-231 cell line treatment for 48 h. (**b**) Cell viability analysis of the MDA-MB-231 cell line treatment for 72 h. * *t*-test: *P*-values of less than 0.05 was considered statistically significant. Our data here, * *P* < 0.01 versus PBS (n = 3).

As shown in Figure 7, PNIPAM-PMAA@Fe_3_O_4_ alone at a higher concentration (0.1 mg/mL) has significant cytotoxicity, whereby only 30% of cells survived. On the other hand, particles loaded with DOX have more of an apparent inhibitory effect, in which merely 16% and 14% of the cells survived, an observation which indicated that doxorubicin could be released after 24 h and thus kill cancer cells. Particles without doxorubicin could kill cells, and this effect increased as particle concentration increased. However, this phenomenon was not desired, as it could limit the application and further development of Fe_3_O_4_-cored MNP. This set of preliminary data points out the concentration effect of the particles. Based on the data obtained, we noticed the importance of improving the drug loading content (amount of drug loaded per milligram of particle) for further applications in cancer therapy.

### 3.9. Cellular Uptake Analysis via Microscopy

**Figure 8:**
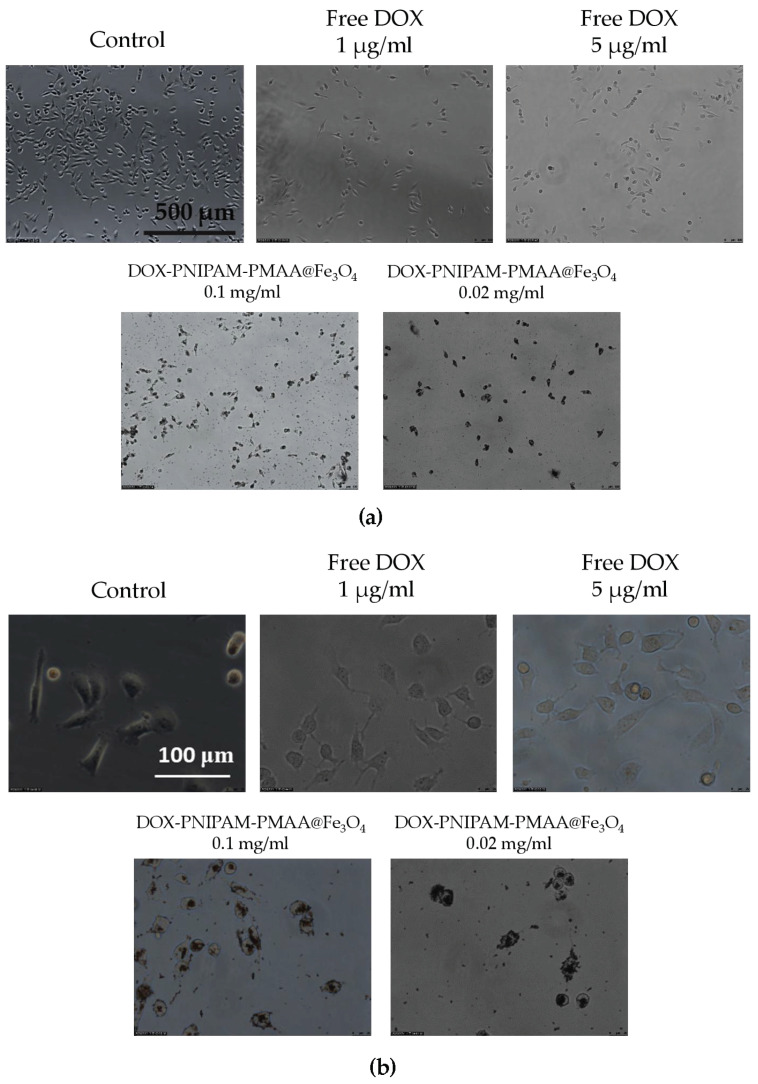
Cellular uptake analysis via microscopy. (**a**) Comparison of particle treatments under bright field observation (100×). (**b**) Comparison of particle treatments under bright field observation (400×).

As shown in Figure 8, it is obvious that whether under 100X or 400X magnification, DOX -PNIPAM-PMAA@Fe_3_O_4_ is significantly seen entering cancer cells (particles were observed in the cells after being washed with PBS several times), and this phenomenon is much more evident under higher DOX and particle concentrations. However, microscopy is limited when observing nanosized subject. Here, a large amount of black-colored DOX-PNIPAM-PMAA@Fe_3_O_4_ was uptaken by the cells, allowing to see the black staining in the cells.

## 4. Discussion

In this series of experiments, numerous methods and analysis means were used to identify whether monomers or RAFT agents were linked on particles. In the first section, we used co-precipitation to form Fe_3_O_4_-cored nanoparticles, and the product was well suspended in water after a sequence of modification. As for the two-step sol-gel method to synthesize NH_2_-SiO_2_@Fe_3_O_4_, 4-nitrobenzaldehyde was used to react with NH_2_-SiO_2_@Fe_3_O_4_, and then, the moiety was cleaved off by ethanolamine. After the reaction, the supernatant became light yellow and was subsequently examined by UV-Vis absorption to estimate the number of amine groups on each unit of particles. Besides, SEM was used to see the actual structures of HO@Fe_3_O_4_ and NH_2_-SiO_2_@Fe_3_O_4._

CPDB@Fe_3_O_4_, PMAA@Fe_3_O_4_, and PNIPAM-PMAA@Fe_3_O_4_ were rather hard to analyze since the particles are made up of iron core, a trait that poses a limitation to many analytical instruments. However, we were still able to identify the differences among these particles through some observed phenomena. Through DLS data, we found that particle hydrodynamic size increased as the monomers were polymerized on the particles. As for zeta-potential analysis, it was clear that after modification of the RAFT agent, the potential of the surface switched from a positive to a negative charge (+16.6 mV for NH_2_-SiO_2_@Fe_3_O_4_ and −20.95 mV for CPDB@Fe_3_O_4_). Furthermore. after polymerization of MAA and NIPAM, the charge differed subtly, as mentioned in the result section. FTIR was also used to observe the monomer distribution of the particles; however, no evident peaks were observed in the spectrum. This may be due to the fact that iron interrupted the transmittance of other moieties and made it difficult for analysis. TGA was applied to determine the organic polymer mass of the PNIPAM-PMAA@Fe_3_O_4._ The pattern of PNIPAM-PMAA@Fe_3_O_4_ it differed from those of CPDB@Fe_3_O_4_ and PMAA@Fe_3_O_4_ with a significant weight loss when the temperature was heated up to 500 °C. Additionally, the total loss amounted to 89% compared to that of PMAA@Fe_3_O_4._ The total loss of PMAA@Fe_3_O_4_ came to 91%, which was 1% higher than CPDB@Fe_3_O_4_. Though we could not accurately estimate the degree of polymerization, the obtained data still verify the thermo-behavior of particles.

Since DLS, zeta-potential, and FTIR analyses may be time consuming for sample preparation, we introduced a rapid method to determine the characteristics and suspensions in different solution. This method is less persuasive because the results are determined through the naked eye; however, there is no doubt that it is an effective and quick method. In the last section, we loaded particles with the anti-cancer drug doxorubicin. Under basic conditions (pH = 8.5), the electrostatic attraction between carboxyl groups and DOX was enhanced, and the result could be estimated by drug loading content and drug encapsulation efficiency. Furthermore, DOX-PNIPAM-PMAA@Fe_3_O_4_ was subjected to fluorescence spectrometry analysis, in which, under 480-nm excitation, we could obviously observe two peaks at 560 and 590 nm. The results indicated that DOX was attached to particles.

Furthermore, the drug release efficacy result matched what we had estimated, because drug release is primarily induced by the acidic microenvironment. In the analysis of DOX release under different temperatures, we also found that higher temperatures could actually enhance drug release efficiency [28]. Thus, we evaluated the release rate under different pH conditions, and as we expected, the maximum drug release rate of particles was observed in the most acidic treatment. Last, we evaluated cell viability and cellular uptake through MTT assay and microscopy observation. As mentioned in the results section, we observed toxicity in the particles without a drug attachment [27], and it was not a positive outcome as it may confine medical applications. We found that after modification of two monomers, the particles could exhibit a cytotoxic property and caused the number of cancer cells to decrease; this effect was dose-dependent. After being loaded with the anti-cancer agent DOX, the anti-cancer effect could be obviously enhanced. Since the modified particles without DOX loading already showed cancer cell inhibition ability, the result might not be very acceptable. However, this could be improved by further modification of the particles surface, such as by polyethylene glycol addition [29]; that is what we will work on in the future. As for the cellular uptake analysis, we could observe that particle scratches and fragments may remain and aggregate in the cells after washing with PBS several times. The results also indicated that with higher concentration, DOX-PNIPAM-PMAA@Fe_3_O_4_ may cause damage to cancer cells and, thus, attain a cancer therapy effect. In this research, we employed co-precipitation method to prepare iron-cored nanoparticles. This method allows users to quickly obtain product, and the yield was quite considerable as well. Furthermore, the method was easy and required no sophisticated instruments compared to other methods [30]. The HO@Fe_3_O_4_ was then modified with an amine group and further polymerization of MAA and NIPAM through RAFT polymerization. The polymerization method is now recognized as a proper way to modify particles since it is more understandable and it could be applied to a wide range of monomers. MAA and NIPAM, the selected monomers, could make the particles dual-responsive. Besides, the iron-cored nanoparticle itself already exhibited magnetic properties. Therefore, the PNIPAM-PMAA@Fe_3_O_4_ could be more functional compared to previous research [31]. This is more prospective and will be developed in the near future. There is more work to be done to improve the particle characterization, as we mentioned in the above contents.

## 5. Conclusions

In this series of experiments, we successfully obtained iron oxide-cored nanoparticles and modified them with both thermal- and pH-sensitive functional groups via RAFT polymerization. DLS, zeta potential, FTIR, and TGA/DSC analyses were performed to determine the characteristics of the particles. Besides, doxorubicin was successfully attached to the particle surface by electrostatic attraction. The releasing effect under an acidic environment was optimal, as we had expected. Cell viability was estimated through the MTT assay. However, the particles seemed to be toxic to cells at a high concentration, and this could be improved by increasing the mass of the encapsulated drug or adjusting the particle mass treated. Finally, microscopy was utilized to perform cellular uptake, and we were amazed to find that particles could enter the cell under bright field observation. To sum up, we have developed an effective vector for biomedical applications. While there is plenty of work and research left to be done, we believe that after further adjustment and modification, iron-cored particles can be a prospective and potential material in the future.

## Figures and Tables

**Table 1:** The analysis of dynamic light scattering (DLS) for particles.

	Size (nm)	S.D.	PDI
HO@Fe_3_O_4_^a^	20.8	4.1	-
NH_2_-SiO_2_@Fe_3_O_4_^a^	282.9	72.8	0.655
CPDB@Fe_3_O_4_^b^	511.0	138.6	0.476
PMAA@Fe_3_O_4_^b^	258.3	59.1	0.313
PNIPAM-PMAA @Fe_3_O_4_^a^	392.8	135.5	0.843

**^a^**: Size distributions of HO@Fe_3_O_4_, NH_2_-SiO_2_@Fe_3_O_4_, and PNIPAM-PMAA@Fe_3_O_4_ were analyzed in dd-H_2_O. **^b^**: Size distributions of CPDB@Fe_3_O_4_ and PMAA@Fe_3_O_4_ were analyzed in DMF.

**Table 2:** Summary of particle suspension conditions in different solvents.

	CPDB@Fe_3_O_4_	PMAA@Fe_3_O_4_	PNIPAM-PMAA @Fe_3_O_4_
Ether	ns	ns	ns
DCM	p	p	p’
H_2_O	s’	s	s
NaHCO_3_	s	s	s

ns: not well suspended; p: precipitation; s’: well suspended but precipitation occurs after a few minute; p’: difficult for separation and precipitaion.

## Data Availability

The data presented in this study are available on request from the corresponding author.

## References

[1] Böhme D., Beck-Sickinger A.G. (2015). Drug delivery and release systems for targeted tumor therapy. J. Pept. Sci..

[2] Yoon H.Y., Koo H., Choi K.Y., Kwon I.C., Choi K., Park J.H., Kim K. (2013). Photo-crosslinked hyaluronic acid nanoparticles with improved stability for in vivo tumor-targeted drug delivery. Biomaterials.

[3] Mody V.V., Cox A., Shah S., Singh A., Bevins W., Parihar H. (2014). Magnetic nanoparticle drug delivery systems for targeting tumor. Appl. Nanosci..

[4] Shubayev V.I., Pisanic T.R., Jin S. (2009). Magnetic nanoparticles for theragnostics. Adv. Drug Deliv. Rev..

[5] Gupta A.K., Wells S. (2004). Surface-modified superparamagnetic nanoparticles for drug delivery: Preparation, characterization, and cytotoxicity studies. IEEE Trans. Nanobiosci..

[6] Whiteside T. (2008). The tumor microenvironment and its role in promoting tumor growth. Oncogene.

[7] Yu X., Yang X., Horte S., Kizhakkedathu J.N., Brooks D.E. (2014). A pH and thermosensitive choline phosphate-based delivery platform targeted to the acidic tumor microenvironment. Biomaterials.

[8] Schmaljohann D. (2006). Thermo- and pH-responsive polymers in drug delivery. Adv. Drug Deliv. Rev..

[9] Li J., Li Y., Wang Y., Ke W., Chen W., Wang W., Ge Z. (2017). Polymer Prodrug-Based Nanoreactors Activated by Tumor Acidity for Orchestrated Oxidation/Chemotherapy. Nano Lett..

[10] Li J.-L., Cheng Y.-J., Zhang C., Cheng H., Feng J., Zhuo R.-X., Zeng X., Zhang X.-Z. (2018). Dual Drug Delivery System Based on Biodegradable Organosilica Core–Shell Architectures. ACS Appl. Mater. Interfaces.

[11] Callahan D.J., Liu W., Li X., Dreher M.R., Hassouneh W., Kim M., Marszalek P., Chilkoti A. (2012). Triple Stimulus-Responsive Polypeptide Nanoparticles That Enhance Intratumoral Spatial Distribution. Nano Lett..

[12] Xu X.-F., Pan C.-Y., Zhang W.-J., Hong C.-Y. (2019). Polymerization-Induced Self-Assembly Generating Vesicles with Adjustable pH-Responsive Release Performance. Macromolecules.

[13] Wang N., Chen X.-C., Ding R.-L., Yang X.-L., Li J., Yu X.-Q., Li K., Wei X. (2019). Synthesis of high drug loading, reactive oxygen species and esterase dual-responsive polymeric micelles for drug delivery. RSC Adv..

[14] Palanikumar L., Kim J., Oh J.Y., Choi H., Park M.-H., Kim C., Ryu J.-H. (2018). Hyaluronic Acid-Modified Polymeric Gatekeepers on Biodegradable Mesoporous Silica Nanoparticles for Targeted Cancer Therapy. ACS Biomater. Sci. Eng..

[15] Hee Kim E., Sook Lee H., Kook Kwak B., Kim B.-K. (2005). Synthesis of ferrofluid with magnetic nanoparticles by sonochemical method for MRI contrast agent. J. Magn. Magn. Mater..

[16] De Biasi F., Rosa-Gastaldo D., Sun X., Mancin F., Rastrelli F. (2019). Nanoparticle-Assisted NMR Spectroscopy: Enhanced Detection of Analytes by Water-Mediated Saturation Transfer. J. Am. Chem. Soc..

[17] Yu S., Gao X., Baigude H., Hai X., Zhang R., Gao X., Shen B., Li Z., Tan Z., Su H. (2015). Inorganic Nanovehicle for Potential Targeted Drug Delivery to Tumor Cells, Tumor Optical Imaging. ACS Appl. Mater. Interfaces.

[18] Riaz S., Bashir M., Naseem S. (2013). Iron oxide nanoparticles prepared by modified co-precipitation method. IEEE Trans. Magn..

[19] Fang J., Nakamura H., Maeda H. (2011). The EPR effect: Unique features of tumor blood vessels for drug delivery, factors involved, and limitations and augmentation of the effect. Adv. Drug Deliv. Rev..

[20] Torchilin V. (2011). Tumor delivery of macromolecular drugs based on the EPR effect. Adv. Drug Deliv. Rev..

[21] Kessenbrock K., Plaks V., Werb Z. (2010). Matrix Metalloproteinases: Regulators of the Tumor Microenvironment. Cell.

[22] Li K., Lu L., Xue C., Liu J., He Y., Zhou J., Xia Z., Dai L., Luo Z., Mao Y. (2020). Polarization of tumor-associated macrophage phenotype via porous hollow iron nanoparticles for tumor immunotherapy in vivo. Nanoscale.

[23] Li H.-Y., Lin H.-C., Huang B.-J., Kai Lo A.Z., Saidin S., Lai C.-H. (2020). Size Preferences Uptake of Glycosilica Nanoparticles to MDA-MB-231 Cell. Langmuir.

[24] Lin H.-C., Hsu K.-F., Lai C.-L., Wu T.-C., Chen H.-F., Lai C.-H. (2020). Mannoside-Modified Branched Gold Nanoparticles for Photothermal Therapy to MDA-MB-231 Cells. Molecules.

[25] Moad G., Chong Y.K., Postma A., Rizzardo E., Thang S.H. (2005). Advances in RAFT polymerization: The synthesis of polymers with defined end-groups. Polymer.

[26] Li Y., Benicewicz B.C. (2008). Functionalization of Silica Nanoparticles via the Combination of Surface-Initiated RAFT Polymerization and Click Reactions. Macromolecules.

[27] Cooperstein M.A., Canavan H.E. (2013). Assessment of cytotoxicity of (N-isopropyl acrylamide) and poly (N-isopropyl acrylamide)-coated surfaces. Biointerphases.

[28] Zheng Y., Wang L., Lu L., Wang Q., Benicewicz B.C. (2017). pH and Thermal Dual-Responsive Nanoparticles for Controlled Drug Delivery with High Loading Content. ACS Omega.

[29] Ebadi M., Buskaran K., Bullo S., Hussein M.Z., Fakurazi S., Pastorin G. (2020). Synthesis and Cytotoxicity Study of Magnetite Nanoparticles Coated with Polyethylene Glycol and Sorafenib–Zinc/Aluminium Layered Double Hydroxide. Polymers.

[30] Lungu I.I., Nistorescu S., Badea M.A., Petre A.-M., Udrea A.-M., Banici A.-M., Fleacă C., Andronescu E., Dinischiotu A., Dumitrache F. (2020). Doxorubicin-Conjugated Iron Oxide Nanoparticles Synthesized by Laser Pyrolysis: In Vitro Study on Human Breast Cancer Cells. Polymers.

[31] Liu X., Hou Y., Zhang Y., Zhang W. (2020). Thermoresponsive Polymers of Poly (2-(N-alkylacrylamide) ethyl acetate) s. Polymers.

